# The relationship between obstructed defecation and true rectocele in patients with pelvic organ prolapse

**DOI:** 10.1038/s41598-020-62376-2

**Published:** 2020-03-27

**Authors:** Cheng Tan, Jing Geng, Jun Tang, Xin Yang

**Affiliations:** 1 Department of Gynaecology, Peking University People’s Hospital, Beijing, China; 2 Beijing Key Laboratory of Female Pelvic Floor Disorders, Beijing, China

**Keywords:** Anatomy, Anal diseases

## Abstract

We aimed to investigate the prevalence of true rectocele and obstructed defecation (OD) in patients with pelvic organ prolapse (POP), to investigate the correlation between true rectocele and OD, and to understand the diagnostic value of translabial ultrasound (TLUS) in the diagnosis of true rectocele. The patients who scheduled for POP surgery were enrolled in this study. Patients who had previous reconstructive pelvic surgery or repair of rectocele were excluded. Birmingham Bowel and Urinary symptoms questionnaires and Longo’s obstructed defecation syndrome scoring system were used to assess the bowel symptoms of patients. TLUS was used to evaluate anatomical defects. P value <0.05 was considered statistically significant, and confidence intervals were set at 95%. 279 patients were included into this study. The prevalence rate of OD was 43%, and the average value of ODS score was 6.67. 17% patients presented straining at stool, 33% presented incomplete emptying, 13% presented digitations, and 12% required laxatives or enema. The prevalence rate of true rectocele was 23%. Defecation symptoms were significantly correlated with age, levator-ani hiatus, levator-ani muscle injury and true rectocele. Logistic regression showed that true rectocele and increased levator-ani hiatus were independent risk factors of OD. True rectocele was significantly correlated with straining at stool, digitation, incomplete emptying and requirement of laxatives or enema.In POP patients, the prevalence rate of true rectocele and OD was 23% and 43%, respectively. True rectocele was related to OD. TLUS was a valuable approach in anatomical evaluation of POP.

## Introduction

Pelvic organ prolapse (POP) is a type of disease caused by the defect of pelvic floor supporting structure, which leads the pelvic organ to leave its own anatomical position. Constipation is a common symptom in POP patients^1^. Previous study has shown that 50% of people with chronic constipation have symptoms of obstructed defecation (OD). OD is characterized by straining at stool, incomplete emptying, digitation (finger extrusion in perineum or vagina to assist defecation) and the requirement of laxatives or enema^2^. Therefore, there should be certain prevalence rate of OD in POP patients. Regarding the relationship between anatomical defects of POP and obstructed constipation, recent studies have suggested that OD is significantly correlated with posterior pelvic anatomic abnormalities, such as true rectocele, enteric hernia and intussusceptions^3^. Some scholars think that posterior vaginal prolapse (PVP) indicates rectocele, while a recent study has shown that true rectocele is only observed in 39% women^4^. The prevalence of true rectocele and its effect on POP patients remain largely unexplored.

Although video-defecography is considered to be the “gold standard” for the diagnosis of rectocele, a number of previous studies have confirmed that translabial ultrasound (TLUS) has good consistency and is in good agreement with video-defecography^5–8^. In the present study, we selected TLUS to evaluate the anatomic abnormalities because of its safety, convenience, better patient-acceptability, and ease of use by gynecologists^4^.

In the present study, we aimed to investigate the prevalence of OD and rectocele in POP patients and evaluate the relationship between OD and rectocele.

## Materials and Methods

This cross-sectional study was approved by the Scientific Committee and the ethics committee of Peking University People’s Hospital (approval number 2015PHB165). All POP patients planning to undergo POP surgery from May 2014 to March 2016 were enrolled in this study. Patients who had previous surgical treatment for POP or rectocele were excluded. Informed consent was obtained from all subjects. All subjects underwent standard evaluation procedures, including demographic data collection, medical history investigation, questionnaire assessment, physical examination and TLUS. The questionnaire employed Birmingham Bowel and Urinary symptoms questionnaire (BBUSQ-22) and ODS scoring system^9–11^. The physical examination included the evaluation of POP using the POP quantification (POP-Q)^12^.

Bowel symptoms were diagnosed based on the patient’s response to BBUSQ-22 (relevant questionnaires were listed in tabular manner in Chart 1). ODS score was the total score of ODS scoring system.

**Figure 1:**
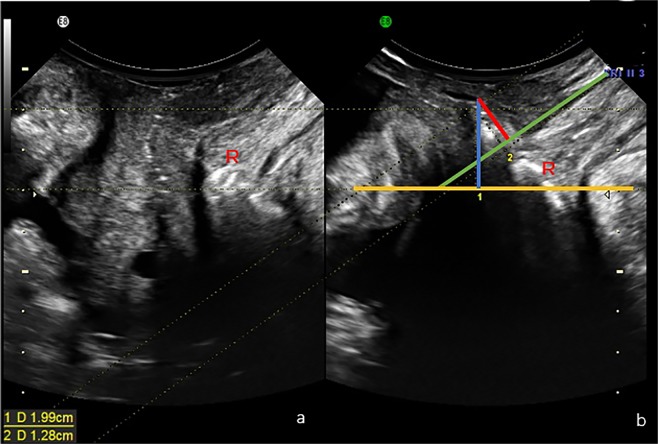
Figures a and b showed the location of the rectum (R) in resting and Valsalva states, respectively. The yellow line refers to the horizontal line from the lower margin of pubis. The green line represents the extended ventral line of internal sphincter. The depth of rectocele was measured by the distance from the farthest point of the ampulla to the extended ventral line of internal sphincter. The rectocele was diagnosed as the discontinuity in the ventral contour of the anorectal muscularis.

The ultrasound assessment was performed according to the current guideline and regulations. The TLUS was performed with patients in the lithotomy position. An abdominal 3-D probe was then placed on the perineum or at the labia with gentle pressure. The bladder was half-filled, and the rectum might be instilled with ultrasound gel. Images were acquired at rest, with contraction and maximal straining^13^. Data of ultrasound volume were blindly analyzed at a later date by the author on a desktop PC using the proprietary software 4D View v 10 (GE Kretz Medizintechnik). POP was determined relative to the posteroinferior margin of the pubic symphysis using volume data acquired on maximum Valsalva, such as in the ultrasound volume demonstrating the most marked POP^14^. An enterocele was diagnosed when the lower margin of the small bowel or omentum reached or was below the pubic bone. A true rectocele was defined as the presence of a discontinuity in the anterior contour of the internal anal sphincter and anterior anorectal muscularis, resulting in a diverticulum of the ampulla, indicative of a defect of the recto-vaginal septum (RVS)^4^. If substantial downwards displacement of the rectal ampulla was seen on image (at least 15 mm below the pubic symphysis) without an actual rectocele, perineal hyper-mobility was diagnosed^15^. Figure 1 illustrates the ultrasound diagnosis of rectocele.

Statistical analysis was carried out with SPSS v12 (IBM Corp., Armonk, NY, USA) and SAS v9.3 (Cary CR: SAS institute INC., USA) for PC. Univariate and multivariate logistic regression analyses were employed to predict symptoms of OD. A p < 0.05 was considered statistically significant.

## Results

A total of 279 patients were enrolled in this study. The mean age of enrolled subjects was 65.4 (SD ± 9.5) years with a mean body mass index (BMI) of 25.2 (SD ± 3.0) kg/m^2^. The median parity was 3 (ranging from 1 to 6). All patients were of Asian ethnicity. Moreover, 62 (22%) and 20 (7%) women previously underwent a hysterectomy and surgery for urinary incontinence, respectively. In addition, 73 (26%) patients reported stress urinary incontinence (SUI), and 34 (12%) patients reported urgency urinary incontinence (UUI).

All patients were diagnosed with stage II or higher degree of prolapse in at least one compartment according to POP-Q classification method. Table 1 lists the POP-Q classification of POP patients.

**Table 1:** POP-Q classification of POP in subjects (total number = 279).

POP-Q staging	anterior compartment	central compartment	Posterior compartment
I	20 (7.2%)	40 (14.3%)	30 (10.7%)
II	71 (25.4%)	63 (22.6%)	126 (45.1%)
III	150 (53.8%)	137 (49.1%)	103 (36.9%)
IV	38 (13.6%)	39 (14.0%)	20 (7.2%)

The prevalence rate of ODS was 43% (120/279), and the average value of ODS score was 6.67. Moreover, 48 (17%) patients presented straining at stool, 92 (33%) presented incomplete emptying, 35 (13%) presented digitations, and 33 (12%) required laxatives or enema.

On TLUS image, a true rectocele was diagnosed in 63 patients (23%) at a mean rectocele depth of 26.7 (SD ± 8.3) mm. Two (0.7%) patients were diagnosed with enterocele. Moreover, 214 (77%) patients were diagnosed with a levator avulsion on tomographic ultrasound imaging (TUI), including 146 (53%) on one side and 68 (24%) on both sides. The mean hiatal area on Valsalva was 34.55 (ranging from 18.1 to 64.5) cm^2^. Two patients were diagnosed with perineal hyper-mobility, and one patient was diagnosed with intussusceptions.

Single factor ANOVA was conducted in order to identify risk factors of OD in POP patients. Results showed that the distribution of patients with different stages of PVP was significantly different between the two groups. Age, levator ani muscle (LAM) injury, hiatal area and the prevalence rate of rectocele were significantly related with OD. The odd ratio of LAM injury was 2.620 (95%CI, 1.417–4.845), and the odd ratio of true rectocele was 19.00 (95%CI, 8.217–43.934) (Table 2)

**Table 2:** Single factor ANOVA of ODS and related factors.

Subject parameter	OD symptoms			
	Yes (n = 120)	No (n = 159)	χ2 or t	P value
Age (year)	67.4 ± 10.3	63.8 ± 6.5	3.566	0.001
BMI (kg/m^2^)	25.4 ± 1.7	25.0 ± 2.6	1.55	0.144
Parity (IQR)	3 (2)	2 (1)	0.91	0.458
Hysterectomy (n,%)	25(21%)	37(23%)	0.235	0.628
PVP* I	13 (11%)	17 (11%)		0.005
II	41 (34%)	85 (53%)		0.264
III	53 (44%)	50 (31%)		0.434
IV	13 (11%)	7 (4%)		0.159
Injury of LAM	yes	103 (86%)	111 (70%)	0.002
	no	17 (14%)	48 (30%)	
Hiatus area	36.2 ± 9.69	33.3 ± 8.57	2.645	0.009
Rectocele	56 (47%)	7 (4%)	69.87	<0.001

*PVP referred to posterior vaginal prolapse.

Multivariate logistic regression analysis showed that age, bilateral LAM injury, blooming of LAM area and rectocele were risk factors for POP patients with OD (Table 3).

**Table 3:** Multivariate logistic regression analysis.

Subject parameter	OD symptoms			
	P-value	OR	Lower bound	Upper bound
Age	0.039	1.315	1.014	1.706
LAM injury	0.016	1.902	1.167	3.361
Enlargement of levator hiatus	0.008	1.548	1.022	2.345
True rectocele	0.002	9.655	3.619	25.796

A comparison between subjects with and without true rectocele revealed that the subjects with true rectocele had higher prevalence rate of straining, digitation, incomplete bowel emptying and requirement of laxatives or enema. The ODS score was significantly different between these two groups(Table 4).

**Table 4:** Analysis of correlation between true rectocele and OD symptoms.

OD symptoms	True rectocele					
	Yes (n = 63, %)	No (n = 216, %)	P-value	OR	95% CI	
					Lower	Upper
straining at stool	35 (55.6%)	13 (6.0%)	0.000	20.481	9.687	43.299
digitation	29 (46.0%)	6 (2.8%)	0.001	29.853	11.536	77.251
incomplete bowel emptying	55 (87.3%)	37 (58.7)	0.000	33.260	14.623	75.651
Laxative or enema to help defecation	12 (19.0%)	21 (9.7%)	0.044	2.185	1.008	4.735
**ODS**						
scoring system	8.5 ± 0.91	6.5 ± 1.67	0.001	NA	NA	NA
**PVP**						
Stage I-II	26 (41%)	130 (60%)	0.008	1.475	1.133	1.921
Stage III-IV	37 (59%)	86 (40%)				

Briefly, 97% patients with true rectocele had II degree or above PVP. Those patients, who had no significant PVP but showed significant rectocele on TLUS, all had history of hysterectomy.

## Discussion

In the present study, we aimed to investigate the prevalence rate of OD and explore the relationship between rectocele and OD in POP patients. In middle-aged women, the prevalence rate of OD is found to be 12.3%, which is related to the history of pelvic floor disorders, and the prevalence rate of OD is increased by 2.3-fold among POP patients possibly due to overlapped risk factors^16,17^.

In our study, the prevalence rate of OD was 43% (120/279) and the average value of ODS score was 6.67. These findings were consistent with previous studies^15,17^. The difference of prevalence rate might be attributed to different diagnostic criteria of OD^18^. Therefore, we call for a unified symptom assessment method for OD.

Meanwhile, pelvic anatomic abnormalities were assessed by TLUS, and 63 (23%) subjects were diagnosed with true rectocele. We found that 56 subjects had true rectocele in the 120 subjects with OD, while only seven subjects had true rectocele in 159 subjects without OD. Statistical analysis showed that the true rectocele was significantly correlated with OD. Four OD-related symptoms were included in the analysis. Moreover, 48 (17%) subjects presented straining at stool, 92 (33%) subjects presented incomplete emptying, 35 (13%) presented digitations, and 33 (12%) required laxatives or enema. We also found that that all four related symptoms were significantly more common in patients with true rectocele. Previous study has also found that incomplete emptying and digitation are most associated with true rectocele^14^.

Guzman Rojas *et al*. have used TLUS to detect anatomic abnormalities in patients of pelvic floor dysfunction (both POP and SUI) and found that more than half of the subjects are diagnosed with true rectocele^19^. The prevalence rate of true rectocele was significantly different from that in our study. The measurement methods in this study were in line with those used in previous studies. The possible reasons for the discrepancies were analyzed as follows. First, the main complaints in this study were POP, and the ODS score was relatively low in this study. Second, the ethnicity of two studies was totally different. Previous study has suggested that the Caucasian ethnicity is a significant factor for posterior compartment prolapse and true rectocele compared with Asian ethnicity^20^. Third, the prevalence rate of OD is higher in Guzman’s study. The prevalence rate of OD is 64% in their study, while the prevalence rate of OD was only 43% in our study. Even though, 44% patients without OD exhibit true rectocele in Guzman’s study, while such rate was only 4% in our study. Further studies are necessary to provide detailed information on the prevalence and risk factors of rectocele in Asian population.

In our present study, in patients with PVP of greater than or equal to II degree, the prevalence rate of true rectocele was 24.5% (61/249), and 97% patients with true rectocele had PVP of greater than or equal to II degree. Some clinicians believe that OD is associated with PVP. However, we found that the patients had severe PVP but not true rectocele, and the average ODS score was 4.3, which was lower than the average level in this study. The average ODS score in patients with true rectocele was 8.5, which was much higher than the average level, indicating that ODS symptoms were associated with rectocele, rather than PVP.

For POP patients, if in combination with OD, TLUS should be performed to evaluate the anatomical defect. In this study, two patients had no significant PVP, but showed significant rectocele on TLUS, and they all had history of hysterectomy (one case was due to atypical endometrial hyperplasia, and the other one was due to multiple uterine fibroids).

Although our study and many other studies support that there is a significant correlation between OD and true rectocele, the causal relationship between OD and rectocele remains unclear.

We found that the diagnosis of rectocele by TLUS was related to OD, indicating a certain value in anatomical evaluation of POP. We also found that PVP was more severe in patients with rectocele, indicating the correlation between true rectocele and the severity of PVP.

There were several advantages in this study. First, a reliable questionnaire was used to assess the subjective symptoms of patients. Second, the anatomic abnormalities were assessed using TLUS, and measurements and analyses were performed by a senior pelvic ultrasound specialist. Third, blinded method was used to ensure the reliability of the results. Fourth, to the best of our knowledge, this study was the first study, which explored the relationship between rectocele and OD in Asian population. There were also several shortcomings in this study. First, the subjects were of Asian ethnicity. Therefore, the conclusions might not be generalized to other races. Second, the subjects of this study were patients with severe prolapse, and could not represent the manifestation of all POP patients. Third, the symptoms of OD are often associated with functional defecation disorders assessed by anorectal manometry^21^. In this study, we didn’t perform anorectal manometry to the patients, so the influence of functional defecation disorders could not be eliminated. Fourth, we didn’t compare TLUS to video-defecography or dynamic MRI which have been used to study posterior compartment abnormalities^22,23^. However, TLUS has been suggested as a first-line investigation for the assessment of patients with OD^24^. To date, several studies have shown moderate to good agreement between TLUS and defecation proctography for the detection of true rectocele, enterocele, and intussusception^6,8,24,25^. Fifth, there were still other anatomic abnormalities, such as internal rectal prolapse, which might be correlated with obstructed defecation^26^. We didn’t include them in this study because of the lack of uniform diagnostic criteria. Further research on the diagnostic value of TLUS are needed.

## Conclusions

In POP patients, the prevalence rate of true rectocele and OD was 23% and 43%, respectively. TLUS showed that true rectocele was related to OD. Collectively, TLUS was a valuable approach in anatomical evaluation of POP.

## Supplementary information

Appendice Chart 1.

## Data Availability

The data sets generated during and analysed during the current study are available from the corresponding author on reasonable request.
