# Supplementary material for: The relationship between obstructed defecation and true rectocele in patients with pelvic organ prolapse
Source: Sci Rep. 2020 Mar 27;10:5599. doi: 10.1038/s41598-020-62376-2 (PMC7101397; doi:10.1038/s41598-020-62376-2)
Supplement: Supplementary file 1 — Appendice Chart 1. [file 41598_2020_62376_MOESM1_ESM.pdf]

The relationship between obstructed defecation and true rectocele in patients with pelvic organ prolapse

Cheng Tan[1,2], Jing Geng[1], Jun Tang[1], Xin Yang[1,2]

Affiliations:

[1]**Department of Gynaecology, Peking University People's Hospital, Beijing, China**

[2]**Beijing Key Laboratory of Female Pelvic Floor Disorders, Beijing, China**

First author: Cheng Tan and Jing Geng contribute equally to this study.

Corresponding author: ①Jun Tang

Contact information: tang_j8181@sina.com

②Xin Yang

Contact information: xinyang_2003@sina.com

Appendice

**Do you have to strain to open your bowels?**	**Never [1]**<br>**Occasionally [2]**<br>**Most of the time [3] ►**<br>**All of the time [4] ►**

**Do you feel that you cannot completely empty your bowel?**	**Never [1]**<br>**Occasionally [2]**<br>**Most of the time [3] ►**<br>**All of the time [4] ►**

**Do you use a finger or pressure to help open your bowels?**	**Never [1]**<br>**Occasionally [2]**<br>**Most of the time [3] ►**<br>**All of the time [4] ►**

**Do you use a finger in your vagina to help open your bowels?**	**Never [1]**<br>**Occasionally [2]**

	**Most of the time** [3] ► **All of the time** [4] ►
**Use of enemas**	**Never** [1] **>1/month, <1/week** [2] **Once a week** [3] ► **Two to three per week** [4] ► **Every defaecation** [5] ►
**Use of enemas**	**Never** [1] **>1/month, <1/week** [2] **Once a week** [3] ► **Two to three per week** [4] ► **Every defaecation** [5] ►

Chart 1 The answer to each question consists of four layers:“no” ,“occasionally”, “most of the time”, and “always”. The diagnosis of straining is based on Q7 “Do you have to strain to open your bowels?”. The diagnosis of incomplete emptying is based on Q9 “Do you feel that you cannot completely empty your bowels?” Equal to or more than "most of the time.” The diagnosis of requirement of laxatives or enema is based on Q4 “Use of laxatives” and Q5 “Use of enema” in ODS scoring system. The

diagnosis of digitation is based on Q10 “Do you use a finger or pressure to help open your bowels?” and Q11 “Do you use a finger in your vagina to help open your bowels?” ►refers to the answer the authors consider positive.
